# Altered Neural Activity in Adolescent Major Depressive Disorder With Nonsuicidal Self-Injury: A Resting-State Functional Magnetic Resonance Imaging Meta-Analysis

**DOI:** 10.1155/np/7885279

**Published:** 2025-11-14

**Authors:** Yanping Shu, Qin Zhang, Zuli Zheng, Yongzhe Hou

**Affiliations:** ^1^Department of Psychiatry of Women and Children, The Second People's Hospital of Guizhou, Guiyang, China; ^2^Department of Radiology, The Second People's Hospital of Guizhou, Guiyang, China

**Keywords:** activation likelihood estimation, adolescent, major depressive disorder, meta-analysis, nonsuicidal self-injury, resting-state functional magnetic resonance imaging

## Abstract

**Background:**

Resting-state functional magnetic resonance imaging (rs-fMRI) reveals diverse neural activity patterns in adolescent major depressive disorder (MDD) with nonsuicidal self-injury (NSSI; nsMDD). However, the reported results are inconsistent. The aim of this study was to conduct a meta-analysis to identify consistent patterns of brain activity alterations in adolescent nsMDD.

**Methods:**

A systematic search was conducted across PubMed, Web of Science, Embase, Google Scholar, Wanfang, and CNKI for rs-fMRI studies that compared nsMDD patients with healthy controls (HCs), up to June 30, 2025. Significant cluster coordinates were extracted for comprehensive analysis. We utilized regional homogeneity (ReHo) and amplitude of low-frequency fluctuations (ALFFs) analyses. Activation likelihood estimation (ALE) was used to identify regions of aberrant spontaneous neural activity in adolescent nsMDD compared to HCs.

**Results:**

Eight studies (249 adolescent nsMDD and 278 HCs) were included. The ALE meta-analysis revealed increased activity in the left lingual gyrus (LING; Brodmann area [BA] 18) in adolescent nsMDD compared to HCs (voxel size = 200 mm^3^; *p* < 0.05). Decreased activity was observed in the right posterior cingulate cortex (PCC; BA 29) in adolescent nsMDD compared to HCs (voxel size = 360 mm^3^; *p* < 0.05). Jackknife sensitivity analyses demonstrated robust reproducibility in five of eight tests for the left LING and in six of eight tests for the right PCC.

**Conclusions:**

This meta-analysis confirms consistent alterations in specific brain regions in adolescent nsMDD, highlighting the potential of rs-fMRI to refine diagnostic and therapeutic strategies.

## 1. Introduction

Major depressive disorder (MDD) is a complex neuropsychiatric condition characterized by pervasive feelings of sadness, pessimism, heightened emotional sensitivity, and cognitive dysregulation [1]. Globally, the prevalence of self-reported depressive symptoms among adolescents was approximately 34% from 2001 to 2020, with a lifetime prevalence of 19% [2]. Adolescence is a critical neurodevelopmental period, highly susceptible to mental disorders like MDD, which can significantly impair academic performance, social functioning, and overall quality of life. Adolescent MDD commonly presents with persistent low mood, anhedonia, irritability, and cognitive impairments, leading to significant disruptions in concentration, interpersonal relationships, and daily functioning [3]. The prevalence of depression in adolescents has been rising steadily. A systematic review revealed that the prevalence of depression among children and adolescents rose from 1989 to 2022, with approximately 21.3% affected globally [3]. Notably, Asia has experienced some of the highest increases, with the prevalence of elevated depressive symptoms among adolescents rising from 24% during 2001–2010 to 37% during 2011–2020. The global point prevalence of MDD in adolescents is estimated to be around 8% [2].

Furthermore, adolescent depression is strongly associated with nonsuicidal self-injury (NSSI), with research indicating that the lifetime prevalence of NSSI among adolescents with MDD was 52%, while the prevalence within the past 12 months was 57% [4]. NSSI is defined as the deliberate and repetitive destruction of one's own body tissue without suicidal intent, commonly manifesting in behaviors such as cutting, scratching, or burning [5]. It is frequently comorbid with a range of psychiatric conditions, particularly in adolescents, and has been classified by the Diagnostic and Statistical Manual of Mental Disorders, Fifth Edition as a condition warranting further clinical research [6, 7]. NSSI is closely linked to emotional dysregulation and is a strong predictor of adverse clinical outcomes, including future suicide attempts [8]. It also significantly impairs psychosocial functioning by disrupting peer relationships, destabilizing family dynamics, and negatively impacting academic and social performance [9]. MDD is a prominent risk factor for the occurrence of NSSI in adolescents, and the severity of depressive symptoms has been associated with a higher likelihood of engaging in NSSI [10, 11]. Research indicates that the incidence of NSSI is markedly elevated among adolescent MDD, with approximately 40.5% engage in NSSI, and females exhibiting higher rates of NSSI [12]. Furthermore, adolescent MDD with NSSI (nsMDD) are at significantly higher risk of suicide attempts compared to those without NSSI behaviors [13]. Additional, adolescent NSSI is linked to a persistently elevated risk of suicide attempts in adulthood [14]. Therefore, research focusing on NSSI play a crucial role in preventing suicide and self-injury among adolescents with depression.

Until now, optimal treatment strategies for adolescents with MDD and NSSI remain under investigation. Psychotherapy and pharmacotherapy may offer some benefit [15]. Dai et al. [16] conducted an 8-week sertraline trial in adolescents with nsMDD and NSSI, demonstrating both efficacy and safety, and further identifying fronto–occipital circuit activity as a potential therapeutic target. Santamarina-Perez et al. [17] examined adolescents with NSSI receiving 4 weeks of psychotherapy and showed that baseline amygdala–prefrontal connectivity strength could predict treatment outcomes. Nevertheless, studies specifically addressing therapeutic approaches in adolescent nsMDD are still limited, and the underlying neurobiological mechanisms remain insufficiently understood.

Resting-state functional magnetic resonance imaging (rs-fMRI) has become a vital tool for investigating the brain's intrinsic functional architecture, providing insights into the disrupted neural circuitry in mental health disorders like MDD [18]. By assessing spontaneous blood oxygen level-dependent signal fluctuations, rs-fMRI reveals patterns of connectivity and activity across brain regions [19], offering a window into the pathophysiology of adolescent nsMDD. Several analytical methods, such as regional homogeneity (ReHo), amplitude of low-frequency fluctuations (ALFF), fractional ALFF (fALFF), and mean ALFF (mALFF), have been developed to assess localized brain activity. ReHo examines local coherence of rs-fMRI signals, ALFF measures the ALFFs to reflect regional functional activity, and fALFF highlights the relative contribution of low-frequency power to overall blood oxygen level-dependent signals. mALFF, which is the mean of ALFF across a region, offers a more stable measure of brain activity by reducing individual voxel fluctuations [20–23]. Despite their effectiveness, findings across studies remain inconsistent, particularly in adolescent nsMDD [16, 24–31]. These discrepancies may be due to differences in study design, sample size, and analytic methods, complicating the identification of reliable neural markers for NSSI in this population.

In light of these inconsistencies, we conducted a systematic meta-analysis using activation likelihood estimation (ALE) to integrate data from multiple rs-fMRI studies. ALE is a meta-analytic technique in neuroimaging, which aggregates peak activation coordinates from different studies to generate spatial probability maps, thereby identifying consistent activation patterns [32]. By synthesizing results across diverse investigations, ALE employs a quantitative approach to highlight convergent brain regions, offering more robust conclusions regarding brain function in specific conditions [33]. A previous ALE study by Jiang et al. [34] identified regions with altered brain activity in the MDD population, but their focus was on individuals with a history of attempted suicide and did not specifically distinguish the adolescent age group.

Accordingly, in this study, we used ALE analysis to integrate and assess data from brain regions with reported abnormalities in prior rs-fMRI studies, including ReHo, ALFF, fALFF, and mALFF. We hypothesize that adolescent nsMDD will exhibit abnormal brain activity compared to healthy controls (HCs), potentially revealing neural mechanisms underlying NSSI. By conducting an ALE meta-analysis, this study aims to identify vulnerable brain regions, thereby enhancing our understanding of the neurobiological basis of nsMDD and its impact on brain function.

## 2. Materials and Methods

### 2.1. Literature Search

The protocol of this ALE meta-analysis has already been registered on the International Platform of Registered Systematic Review and Meta-analysis Protocols (Registration Number: CRD42024618347).

A systematic search for relevant studies was conducted in the PubMed, Web of Knowledge, Embase, Google, Wanfang, and CNKI databases up to June 30, 2025. Keywords included (“adolescent” OR “youngsters” OR “juvenile”) AND (“depression” OR “depression neurosis” OR “depressive disorder” OR “melancholia” OR “major depression”) AND (“nonsuicidal self-injury” OR “NSSI” OR “Self-Injurious Behavior” OR “Self Harm” OR “Self Destructive”) AND (“regional homogeneity” OR “ReHo” OR “amplitude of low-frequency fluctuation” OR “ALFF”) AND (“magnetic resonance” OR “MRI” OR “functional MRI” OR “fMRI”). In addition, references to included studies and relevant reviews were searched as a supplement.

### 2.2. Eligibility Criteria and Data Extraction

**Figure 1:**
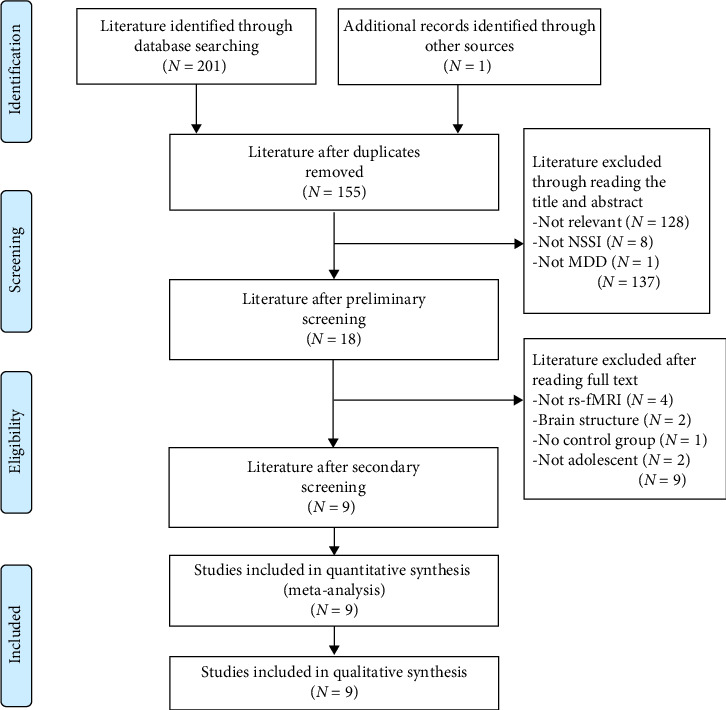
The flow diagram of the search strategy and retrieved studies according to the PRISMA guidelines. MDD, major depressive disorder; *N*, number; NSSI, nonsuicidal self-injury; PRISMA, preferred reporting items for systematic reviews and meta-analyses; rs-fMRI. resting-state functional magnetic resonance imaging.

Studies were considered eligible based on the following criteria: (1) Participants were diagnosed with MDD according to standardized diagnostic criteria (e.g., DSM-IV or DSM-5); (2) the study included adolescent participants; (3) NSSI was clearly defined in the study; (4) rs-fMRI data were analyzed using whole-brain voxel-wise methods; (5) activation coordinates were reported in standard stereotactic space (e.g., Montreal Neurological Institute [MNI] or Talairach coordinates). Exclusion criteria included the following: (1) Inclusion of participants with comorbid psychiatric disorders (e.g., bipolar disorder, schizophrenia, or anxiety disorders); (2) studies not specifically addressing NSSI (e.g., focused on suicidal ideation or suicide attempts); (3) nonoriginal articles (e.g., reviews, case reports, or meta-analysis); (4) studies using non-rs-fMRI methods (e.g., task-based or structural MRI); or (5) studies for which the full text was unavailable or stereotactic coordinates were not reported. Based on the reporting approaches of previous studies [35, 36], we enhanced the transparency of study selection in accordance with the PRISMA 2020 [37] guidelines (Figure 1), and the corresponding checklist is provided as supporting information.

Two authors (Yanping Shu and Yongzhe Hou) individually extracted data from each included study to enhance transparency and scientific rigor. Any discrepancies during the extraction process were resolved through discussion to reach consensus. The extracted information included bibliographic details, sample size, age, sex, years of education, diagnostic criteria for MDD and NSSI, medication status of nsMDD patients, and depression rating scale scores. For rs-fMRI data, we collected information on MRI scanner type and field strength, analytical methods used (e.g., ALFF, fALFF, mALFF, and ReHo), the number of brain regions showing significant differences between adolescents with nsMDD and HCs, statistical correction methods, and the stereotactic space used for reporting results. All coordinate data were independently extracted in accordance with the requirements of ALE meta-analysis.

As different rs-fMRI analytical methods capture distinct aspects of spontaneous neural activity, their combined use may introduce methodological heterogeneity, which should be considered when interpreting the results. Future studies should aim to standardize analytical approaches to improve comparability and reproducibility.

### 2.3. Quality Assessment

The quality of the included studies was assessed by using the Newcastle Ottawa Quality Assessment Scale (NOS) [38]. The NOS has three levels and a total of eight items: (1) four items for subject selection; (2) one item for comparability between groups; (3) three items for outcome measurement. The total score is 9 points. A result ≥5 points was included in the data analysis. The retrieved records were imported into Endnote (X9). After removing duplicates, two researchers (Yanping Shu and Yongzhe Hou) independently eliminated irrelevant records by reading the titles and abstracts, then screened the rest records in full text to identify eligible studies. After selection, two reviewers cross-checked, and disagreements were settled through team discussion or consultation with the third reviewer (Qin Zhang).

### 2.4. ALE Analysis

The ALE approach is a coordinate-based meta-analysis [39] and, as such, consists in taking the activation foci extracted from the records of interest and creating a probability distribution around the reported peak. The algorithm then generates ALE maps by testing the null hypothesis that activation foci are uniformly spread across the whole brain, under the assumption that each voxel has the same probability of being activated. Thus, ALE maps are obtained by computing the union of activation probabilities for each voxel, returning a map of significant spatial convergence among the contrasts of interest.

We used the GingerALE 3.0.2 software (http://brainmap.org/ale) to conduct these analyses. First, all coordinates were automatically converted to MNI using GingerALE Talaraich to MNI (SPM) built in converter. Then, the software computes the full-width at half maximum (FHWM) describing the uncertainty of spatial location (i.e., the probability distribution) of each focus, for each experiment. Afterwards, model activation maps are computed for each voxel by taking the voxel-wise union of the modeled probability values of all foci, for each experiment. These model activation maps represent the summary of the results reported in a specific study considering the spatial uncertainty associated with the reported foci coordinates. ALE scores are then computed as the union of these probabilities across experiments for each. These scores are tested against the null distribution calculated for each voxel, reflecting a random spatial association of the model activation across experiments [40]. The resulting thresholded ALE map was then computed using a false discovery rate (FDR) correction with a *p* value = 0.001 and a minimum cluster size of 10 mm^3^ [41]. Automated anatomical labeling atlas 3 [42] and Human Connectome Project [43] functional atlases had been used to describe the results of the present meta-analyses. Finally, our study used Mango software (http://rii.uthscsa.edu/mango/) to check and analyze the resulting ALE images. In addition, we did not conduct subgroup analyses due to the limited number of studies.

### 2.5. Sensitivity Analysis

The jackknife sensitivity analysis method was employed to assess the reproducibility of the meta-analysis outcomes [44]. In this approach, a single study was systematically excluded from the dataset, followed by ALE meta-analysis of the remaining study data using GingerALE 3.0.2 software. This procedure was repeated 8 times (each time removing one study) to verify the consistency of the results after the exclusion of a study and to compare these results with the original analysis.

## 3. Results

### 3.1. Included Studies and Sample Characteristics

The systematic search identified 202 relevant articles, with one additional study included from the references of the retrieved articles, resulting in nine studies [16, 24–31] being selected for inclusion in this meta-analysis (Figure 1). These studies, published between 2022 and 2024, focused on adolescents with a mean age of 16.17 ± 3.00 years, representing the early to late-adolescent stages of development. Ultimately, nine eligible investigations were included in the meta-analysis, employing various rs-fMRI analysis techniques, including ReHo, ALFF, mALFF, and fALFF. Altogether, 249 adolescents with nsMDD and 278 HCs were included, with 66 distinct brain regions undergoing ALE meta-analysis. The nsMDD group was characterized by a predominantly female composition (83.1%), an average illness duration of 20.6 months, and a mean educational attainment of 9.3 years, in contrast to the HC group with 75.9% female participants and 9.5 years of education on average, with all nsMDD individuals being drug-naïve at the time of scanning, thereby minimizing potential pharmacological confounds (Table 1).

According to the NOS, all included studies showed low publication bias and met the quality criteria for data analysis.

### 3.2. ALE Meta-Analysis Results

**Figure 2:**
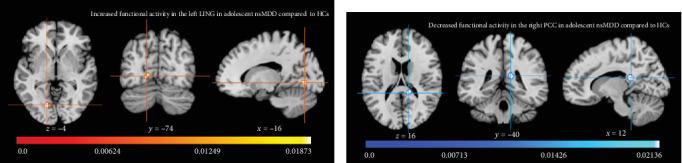
Significant brain activity abnormalities between adolescent nsMDD and HCs. (a) Increased functional activity (warm color) in the left LING (BA 18). (b) Decreased functional activity (cold color) in the right PCC (BA 29). nsMDD, major depressive disorder with nonsuicidal self-injury. BA, Brodmann area; HCs, healthy controls; L, left; LING, lingual gyrus; PCC, posterior cingulate cortex; R, right.

The ALE meta-analysis identified significant alterations in brain activity in adolescents with nsMDD compared to HCs (Table 2 and Figure 2). In Figure 2a, increased activity was observed in the left lingual gyrus (LING; Brodmann area [BA] 18; MNI: −16, −74, −4; ALE value = 0.019; *Z* = 5.62; voxel size = 200 mm^3^; *p* < 0.001). In Figure 2b, decreased activity was found in the right posterior cingulate cortex (PCC; BA 29; MNI: 12, −40, 16; ALE value = 0.021; *Z* = 6.03; voxel size = 360 mm^3^; *p* < 0.001). No significant differences were observed in other brain regions.

**Figure 3:**
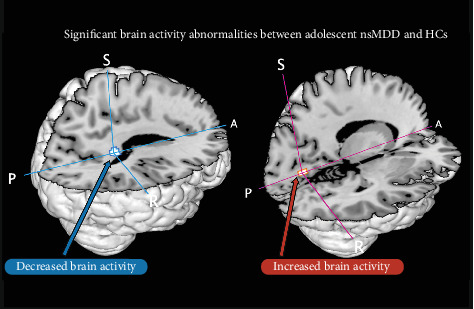
Three-dimensional surface rendering of altered functional activity in adolescent nsMDD compared to HCs. Red arrow indicates increased functional activity in the left LING (BA 18). Blue arrow indicates decreased functional activity in the right PCC (BA 29). nsMDD, major depressive disorder with nonsuicidal self-injury. A, anterior; HCs, healthy controls; LING, lingual gyrus; P, posterior; PCC, posterior cingulate cortex; R, right; S, superior.

To better visualize the spatial distribution of these alterations, a 3D surface rendering of the significant clusters was generated (Figure 3).

### 3.3. Sensitivity Analysis

The sensitivity analysis provided additional insight into the robustness and reproducibility of the findings. In the Jackknife analysis of increased brain activity in adolescent nsMDD, the left LING was consistently identified in five out of eight iterations. The corresponding ALE values ranged from 0.0163 to 0.0187, and the cluster volumes ranged from 64 to 360 mm^3^. Similarly, for decreased brain activity, the right PCC was consistently observed in six out of eight iterations, with ALE values ranging from 0.0178 to 0.0214 and cluster volumes ranging from 16 to 416 mm^3^. While some variability was noted across iterations, the overall pattern suggests a relatively consistent involvement of these regions (Table 3).

## 4. Discussion

To the best of our knowledge, this is the inaugural ALE–based meta-analysis focusing on rs-fMRI brain imaging in adolescents with nsMDD. Using the ALE method, we integrated multiple neuroimaging studies on brain function in adolescent nsMDD to explore potential neurobiological mechanisms. By examining dynamic changes in resting-state brain activity, we specifically combined findings from studies employing ReHo, ALFF, fALFF, and mALFF to identify brain regions where activity differed significantly between adolescent nsMDD and HCs. Our analysis revealed increased brain activity in the left LING and decreased activity in the right PCC in adolescent nsMDD compared to HCs. This convergence of evidence suggests the robustness of our findings. Jackknife sensitivity analyses further indicated a relatively consistent pattern across most iteration, lending cautious support to the stability of the observed differences. These findings may help identify potential therapeutic targets for adolescent nsMDD.

### 4.1. Findings of Brain Regions With Increased Spontaneous Neural Activity in Adolescent nsMDD

The regions showing increased activation, particularly in the left LING (BA 18), are key components of the visual network, which is closely linked to emotional and cognitive processing [45]. Situated in the occipital lobe, the LING is involved in visual perception, sensory integration, and memory encoding, and is essential for processing emotional stimuli and organizing complex visual information [46, 47].

The observed increase in spontaneous neural activity in the left LING among adolescents with nsMDD may reflect aberrant engagement of sensory processing pathways, potentially serving as a compensatory response to the emotional dysregulation and self-injurious tendencies commonly observed in this population. Previous studies have suggested that increased activation in the LING in MDD patients may indicate difficulties in emotion processing and has been associated with suicidal behavior [48]. Furthermore, Dai et al. [16] found that adolescents with nsMDD exhibited increased mALFF in the left LING, speculating that this heightened resting-state activity in the LING was associated with NSSI behavior, underscoring a compensatory rather than efficient neural response. In this study, we found that adolescent nsMDD exhibited increased spontaneous neural activity in the left LING compared with HCs, which was consistent with previous studies. This aberrant activation may signify dysfunctional emotion–cognition integration, which could compromise inhibitory control and exacerbate self-referential negative processing, thereby heightening the risk of engagement in self-injurious behavior [49]. Dysfunction within the visual network, involving the LING, may partly account for this disrupted integration [50]. However, whether such functional alterations represent a neurobiological vulnerability predisposing individuals to NSSI, or are secondary to repeated self-injurious behavior, remains to be determined. Longitudinal neuroimaging studies will be necessary to clarify the temporal and causal relationships underlying these findings.

Besides, the LING plays a critical role in emotional processing, especially in response to negative emotion [51], with additional studies indicating that adolescent MDD show increased abnormal neural activity in the left LING, which is associated with negative processing bias [52]. This may explain the heightened sensitivity to negative emotions and distorted emotional processing in individuals with depression during adolescence [53], a critical developmental period characterized by ongoing brain maturation and emotional reactivity [54], potentially amplifying distressing emotional experiences and influencing maladaptive behaviors like NSSI. Moreover, one study reported that, compared to the MDD without NSSI group, the nsMDD group exhibited higher ALFF in the right LING, with ALFF values positively correlated with the frequency and severity of NSSI in depression patients (*r* = 0.458; *p*=0.0005; Bonferroni corrected) [55]. Another study found that alterations in the surface area of the right LING correlate with the severity of anxiety–depression symptoms [56]. You [28] found a significant positive correlation between ALFF values in the left LING and scores on the self-rating anxiety scale and self-rating depression scale in adolescent nsMDD. These findings suggest that the LING, as part of the visual network involved in visual perception and emotional processing [57], may amplify visual stimuli and memory related to NSSI. Increased resting-state activity in the LING may heighten sensitivity to emotional and visual cues, potentially triggering self-harming behaviors.

Additionally, it has been demonstrated that after sertraline treatment, mALFF in the bilateral LING decreases in adolescent with nsMDD, which may indicate that abnormal activation in the occipital cortex is alleviated following treatment [24]. This improvement is hypothesized to result from sertraline's ability to increase the concentration of gamma-aminobutyric acid in the occipital cortex of depression patients. With the increase in gamma-aminobutyric acid levels, a subsequent reduction in neuronal activity in the occipital cortex may occur [58]. These findings suggest that the Ling may hold therapeutic potential as anerobiological target in adolescents with nsMDD. The observed increase in spontaneous neural activity in the left LING supports the hypothesis that visual network dysfunction contributes significantly to the neuropathology of adolescent depression accompanied by NSSI behaviors.

Given the role of the LING in visual processing and emotional perception—functions frequently disrupted in nsMDD—its aberrant activation may serve as a potential early biomarker for identifying high-risk adolescents. Further investigation is warranted to determine whether modulation of this region, through interventions such as deep brain stimulation or transcranial magnetic stimulation, could improve visual-affective integration and enhance emotional regulation. While preliminary, such findings may eventually contribute to the development of biomarker-informed approaches for early diagnosis and individualized treatment in adolescent nsMDD.

### 4.2. Findings of Brain Regions With Decreased Spontaneous Neural Activity in Adolescent nsMDD

Our ALE meta-analysis also observed decreased spontaneous neural activity in the right PCC (BA 29) in adolescents with nsMDD compared to HCs. The PCC, a core region of the default mode network, is critical for emotional regulation, self-referential thinking, and the integration of cognitive and affective information [59]. The default mode network is closely linked to MDD, potentially disrupting self-recognition and emotional regulation [60]. Ding et al. [61] conducted an ALE meta-analysis, revealing reduced activity in regions of the default mode network in adolescent MDD, suggesting that compromised default mode network integrity is a phenomenon frequently observed in depression. Previous studies have found abnormal brain network function in patients with MDD. Ho et al. [62] discovered that depression patients with NSSI exhibit abnormalities in the default mode network and central executive network. Similarly, Huang et al. [63] observed reduced functional connectivity between the right fusiform gyrus and bilateral PCC. They suggested that this disruption in default mode network functional integration may underlie the neural mechanisms driving NSSI behavior in MDD.

Dysfunction in the PCC has been associated with self-harm and suicidal behavior, which are important features of MDD [64]. One study found that aberrant functional connectivity between the left PCC and left inferior frontal gyrus was associated with increased severity of suicidal ideation in MDD, suggesting its role in rumination, negative self-evaluation, and maladaptive coping behaviors [65]. Another study found abnormal functional connectivity in the left medial gyrus and left lateral cingulate gyrus in adolescents with NSSI, indicating functional abnormalities that may serve as potential pathological mechanisms underlying the risk of self-injurious impulses and impaired inhibitory control [28]. Zhang et al. [66] found that adolescents with MDD exhibited hypoconnectivity in the right PCC compared to HCs, which may contribute to memory deficits and underlie emotional dysregulation and abnormalities in self-referential processing [67]. Impaired emotional regulation is considered a key factor in the development of NSSI in adolescents [68]. These difficulties in memory retrieval and self-reflection may, in turn, increase the risk of NSSI by exacerbating impulsivity and maladaptive coping strategies [69].

Furthermore, research consistently demonstrated that adolescents with NSSI exhibited cognitive difficulties, such as rumination, which were associated with reduced activation in the posterior cingulate [70]. An fMRI study found that reduced neural responses in the PCC of NSSI patients, compared to HCs, were significantly correlated with rejection sensitivity (*r* = 0.42; *p*=0.04), implying perturbations in emotional regulation and self-referential processing during social evaluation [71]. A prior fMRI meta-analysis indicated that heightened activity in both the anterior and posterior cingulate cortices could be implicated in the manifestation of suicidal behavior [72]. Jiang et al. [34] observed that individuals with suicide attempts displayed decreased functional activity in the bilateral PCC. Recent research has elucidated the structural abnormalities within the PCC in adolescent MDD patients. For instance, studies have reported that the right PCC gray matter volume is reduced in adolescent MDD compared to HCs and mediation analysis has found that the right PCC may play a protective role in psychological resilience [73]. Given the role of the PCC in affective self-referential processing and its central position within the default mode network, altered activity in this region may reflect early disruptions in neural systems responsible for emotion regulation and introspective awareness. These changes could precede overt clinical symptoms or suicidal behaviors, offering potential utility for early identification of high-risk adolescents. Therefore, the observed hypoactivity in the right PCC may serve as a promising early biomarker for adolescent depression accompanied by NSSI.

Taken together, the decreased spontaneous neural activity in the right PCC may reflect dysfunctions in emotion regulation and self-reflection processes in adolescents with nsMDD, indicating potential impairments in the default mode network. Identifying these vulnerable regions provides valuable insights into the neurobiological underpinnings of nsMDD. The consistent involvement of the PCC across studies suggests it may serve as an early neuroimaging biomarker to assist in clinical risk stratification and timely intervention. Moreover, these findings support the notion that PCC dysfunction may precede the emergence of overt clinical symptoms or suicidal behaviors, underscoring its potential as an early neuroimaging biomarker. This may hold clinical utility in identifying high-risk adolescents and guiding timely, individualized interventions aimed at preventing the progression of depressive pathology and NSSI behaviors.

### 4.3. Explanations for Selective Brain Activity Abnormalities

Our ALE meta-analysis revealed selective abnormalities, with significant alterations in the left LING and right PCC, but no notable changes in other brain regions. This pattern may be partly explained by the relatively small number of activation foci—only 66 reported across the included studies—available for statistical convergence. The nine studies employed different rs-fMRI analysis methods (e.g., ReHo, ALFF, fALFF, and mALFF), each with inherent technical differences that could introduce biases in detecting brain activation [74]. Additionally, the coordinates reported in these studies may have been influenced by variables such as sex, disease duration, and severity of NSSI symptoms. Consequently, while consistent activity changes were observed in certain brain regions, the heterogeneity across studies may have limited the identification of abnormal activity in other areas. This heterogeneity may also help explain the partial consistency observed in the jackknife sensitivity analysis, where the left LING was significant in five of eight iterations and the right PCC in six of eight, indicating that methodological and sample-related variations likely influenced the stability of the results.

It is interesting to note that only the LING and PCC showed significant changes, which may be explained by their involvement in key functional networks. The LING, as part of the visual network, is crucial for emotion regulation and visual information integration, and abnormalities in adolescent depression have been linked to hemispheric asymmetries in this network [75]. Similarly, the PCC is a core hub of the default mode network, strongly involved in self-referential thought and emotion regulation, with recent evidence suggesting hemispheric imbalance in emotional processing [76]. Therefore, the selective abnormalities in the LING and PCC may reflect disrupted spontaneous brain activity within these networks in adolescents with nsMDD.

Moreover, the ALE meta-analysis, which constructs probability distributions, is more strongly influenced by studies that report a larger number of activation foci [77]. The scarcity of coordinates may fail to meet the statistical threshold criteria, or the spatial distribution of these foci may have been too diffuse, preventing the consolidation of significant results in regions adjacent to the activation points [78]. Such limitations may also help explain the partial consistency seen in the jackknife analysis, suggesting that variability in study design and analytical methods could have influenced result stability. This spatial dispersion could also explain why decreased activity was observed in the right PCC, where the volume of the affected area was only 360 mm^3^, and increased activity was observed in the left LING, where the volume of the affected area was only 200 mm^3^.

In our previous study on adolescents and young adults with MDD, we identified structural abnormalities in brain regions such as the right caudate head, right insula, and right lentiform nucleus putamen [79], suggesting that structural and functional abnormalities may not always align in MDD. Therefore, whether other specific brain regions in adolescents with nsMDD exhibit altered activity relative to HCs, and whether subgroup analyses could uncover such abnormalities, warrants further investigation.

### 4.4. Limitations

We should underline several limitations in our study. First, all included studies were conducted in Asian populations, which may restrict the generalizability of our findings to other cultural or ethnic groups. Cultural and biological factors could influence both NSSI behaviors and their neural correlates; therefore, future multicenter studies across more diverse populations are needed to validate and extend these results. Second, methodological variations in the rs-fMRI metrics employed across studies (e.g., ALFF, fALFF, mALFF, and ReHo) may have introduced heterogeneity, as these indices capture different aspects of spontaneous neural activity. Such differences could affect the pooled results and should be considered when interpreting the findings. Third, several included studies had relatively small sample sizes [16, 28], which may compromise statistical power and result stability. Although the jackknife sensitivity analysis supported the robustness of our results, future investigations with larger and more representative samples are warranted. Finally, from the perspective of the meta-analysis, while the ALE methodology is effective in minimizing the risk of false-positives, it is less capable of addressing false-negatives, which may limit the robustness of the findings [80].

## 5. Conclusion

In summary, this study employed the ALE method to investigate adolescent nsMDD, revealing abnormal spontaneous neural activity in the right PCC and left LING during resting-state compared to the control group. These findings suggest that the altered neural activity in these regions may be closely related to the pathophysiology of NSSI in adolescents with nsMDD. This provides support for further investigation into whether these neural abnormalities are a consequence of NSSI or if individuals with these neural characteristics are more predisposed to developing NSSI behaviors. Future research should focus on the neurobiological mechanisms underlying these abnormalities and develop more targeted clinical strategies for managing NSSI in this population.

## Figures and Tables

**Table 1:** Demographic and clinical characteristics of the studies included in meta-analysis.

Author (year)	nsMDD						HCs			MRI equipment and field strength	Method	Differential brain region	Corrective methods	Coordinate system	Quality score
	Sample size, female *n* (%)	Age (Y)	Education (Y, mean ± SD)	Illness duration (months, mean ± SD)	Depression score	Diagnostic criteria (MDD/NSSI)	Sample size, female *n* (%)	Age (Y)	Education (Y, mean ± SD)						
Zhou et al. (2022) [24]	25, 20 (80%)	14.48 ± 1.36	8.72 ± 1.65	20.08 ± 26.01	NA	DSM-IV/DSM-V	25, 19 (76%)	14.96 ± 1.43	8.96 ± 1.77	GE Signa HDxt 3.0T	ALFF/fALFF/ReHo	19	GRF, *p* < 0.05	MNI	4/1/1
Liang (2022) [25]	36, 30 (83%)	15.22 ± 1.46	9.14 ± 1.55	NA	25.08 ± 5.15^a^	DSM-V/OSI	30, 22 (73%)	14.50 ± 1.96	8.70 ± 2.00	Siemens Trio Tim 3.0T	mALFF	7	GRF, *p* < 0.05	MNI	4/1/1
Xin et al. (2022) [26]	27, 17 (63%)	13.59 ± 2.41	6.30 ± 2.38	6.96 ± 1.22	24.33 ± 1.86^a^	DSM-V/DSM-V	50, 33 (66%)	13.76 ± 2.53	6.60 ± 2.72	Siemens Trio Tim 3.0T	ALFF	3	GRF, *p* < 0.05	MNI	4/1/1
Dai et al. (2023) [16]	15, 13 (87%)	14.60 ± 1.35	9.00 ± 1.77	NA	23.53 ± 3.66^b^	DSM-V/DSM-V	22, 13 (59%)	15.27 ± 1.05	9.36 ± 2.72	GE Signa HDxt 3.0T	mALFF	4	FWE, *p* < 0.05	MNI	4/1/1
Dai (2023) [27]	24, 21 (88%)	14.42 ± 1.28	8.79 ± 1.50	15.42 ± 13.65	22.04 ± 3.67^b^	DSM-IV/DSM-V	22, 13 (59%)	15.27 ± 2.05	9.36 ± 2.72	GE Signa HDxt 3.0T	ALFF/ReHo	6	FWE, *p* < 0.05	MNI	4/1/1
You (2023) [28]	15, 13 (87%)	14.87 ± 0.74	9.6 ± NA	NA	79.40 ± 2.56^c^	DSM-V/DSM-V	15, 12 (80%)	14.67 ± 0.82	9.4 ± 1.50	Siemens Trio Tim 3.0T	ALFF/ReHo/fALFF	9	GRF, *p* < 0.05	MNI	4/1/1
Huang (2023) [29]	30, 25 (83%)	15.80 ± 1.30	9.80 ± 1.30	18.30 ± 11.8	20.50 ± 6.00^b^	DSM-V/DSM-V	28, 23 (82%)	15.50 ± 1.50	9.50 ± 1.50	Siemens Trio Tim 3.0T	ReHo/fALFF	2	GRF, *p* < 0.05	MNI	4/1/1
Huang et al. (2024) [30]	54, 48 (89%)	20.80 ± 3.60	13.00 ± 2.20	42.00 ± 36.00	22.80 ± 5.15^b^	DSM-V/DSM-V	66, 58 (88%)	21.10 ± 2.00	13.60 ± 1.50	Siemens Trio Tim 3.0T	fALFF/ReHo	6	GRF, *p* < 0.05	MNI	4/1/1
Bo and Miao (2024) [31]	23, 20 (87%)	15.13 ± 2.28	9.21 ± 2.13	NA	26.78 ± 5.61^b^	DSM-V/DSM-V	20, 18 (90%)	15.75 ± 1.29	9.70 ± 0.98	Siemens Trio Tim 3.0T	ALFF/ReHo/fALFF	10	FWE, *p* < 0.001	MNI	4/1/1

*Note:* nsMDD, nonsuicidal self-injury major depressive disorder.

Abbreviations: ALFF, amplitude of low-frequency fluctuation; DSM-V, Diagnostic and Dtatistical Manual of Mental Disorders; fifth edition; F, female; fALFF, fractional ALFF; FWE, family-wise error; GRF, Gaussian random field; HCs, healthy controls; M, months; mALFF, mean ALFF; MNI, Montreal Neurological Institute; MRI, magnetic resonance imaging; NA, not available; OSI, Ottawa Self-Injury Inventory; ReHo, regional homogeneity; Y, years.

^a^Hamilton Depression Rating Scale-24 items (HAMD-24).

^b^Hamilton Depression Rating Scale-17 items (HAMD-17).

^c^Self-rating depression scale (SDS).

**Table 2:** Results of ALE meta-analysis between adolescents with nsMDD and HCs.

Research methods	Anatomical label	Brodmann area	Peak MNI coordinate			ALE value	*Z*-score	*p*-Value	Volume (mm^3^)
			*X*	*Y*	*Z*				
Increase									
ReHo and ALFF/mALFF/fALFF	LING.L	BA 18	−16	−74	−4	0.01873446	5.6208396	9.50 × 10^−9^	200
Decrease									
ReHo and ALFF/mALFF/fALFF	PCC.R	BA 29	12	−40	16	0.02135998	6.0321746	8.09 × 10^−10^	360

Abbreviations: ALE, activation likelihood estimation; ALFF, amplitude of low-frequency fluctuation; BA, Brodmann area; fALFF, fractional ALFF; L, left; LING, lingual gyrus; mALFF, mean ALFF; MNI, Montreal Neurological Institute; PCC, posterior cingulate cortex; R, right; ReHo, regional homogeneity.

**Table 3:** Jackknife sensitivity analyses.

Excluded study	Adolescent nsMDD > HCs			Adolescent nsMDD < HCs		
	LING.L	ALE value	Volume (mm^3^)	PCC.R	ALE value	Volume (mm^3^)
Zhou et al. [24]	Yes	0.018733118	360	Yes	0.02135998	384
Liang [25]	N/A	N/A	N/A	Yes	0.02135998	416
Xin et al. [26]	Yes	0.01873446	232	Yes	0.02135998	376
Dai et al. [16]	No	No (N/A)	No (N/A)	Yes	0.02135998	376
Dai [27]	No	No (N/A)	No (N/A)	Yes	0.019896476	224
You [28]	No	No (N/A)	No (N/A)	No	No (N/A)	No (N/A)
Huang [29]	Yes	0.01873446	232	N/A	N/A	N/A
Huang et al. [30]	Yes	0.01627312	64	No	No (N/A)	No (N/A)
Bo and Miao [31]	Yes	0.01873446	272	Yes	0.017758502	16
Total	5 out of 8	—	—	6 out of 8	—	—

*Note:* nsMDD, nonsuicidal self-injury major depressive disorder.

Abbreviations: ALE, activation likelihood estimation; HCs, healthy controls; L, left; LING, lingual gyrus; N/A, not applicable; PCC, posterior cingulate cortex; R, right.

## Data Availability

The data that support the findings of this study are available from the corresponding author upon reasonable request.

## Supporting Information

Supporting Information PRISMA Checklist. This supporting information includes the PRISMA checklist, ensuring that the meta-analysis in this manuscript adheres to the reporting standards for systematic reviews.
